# Data Access for the Open Access Literature: PLOS's Data Policy

**DOI:** 10.1371/journal.pbio.1001797

**Published:** 2014-02-25

**Authors:** Theodora Bloom, Emma Ganley, Margaret Winker

**Affiliations:** 1 Editorial Director for Biology, *PLOS Biology*, Public Library of Science, Cambridge, United Kingdom; 2 Senior Editor, *PLOS Biology*, Public Library of Science, Cambridge, United Kingdom; 3 Senior Research Editor, *PLOS Medicine*, Public Library of Science, San Francisco, California, United States of America

## Abstract

The new PLOS Data Policy will require all submitting authors to include a data availability statement as of March 1, 2014. Read more about what this means and see the policy itself in this cross-PLOS Editorial from Theo Bloom and colleagues.

This Editorial has been published simultaneously in PLOS Biology and PLOS Medicine and is authored by editors from both journals. The Data Policy applies to all PLOS journals.

## Background

Data are any and all of the digital materials that are collected and analyzed in the pursuit of scientific advances. In line with its stance on providing Open Access to research articles themselves, PLOS strongly believes that, to best foster scientific progress, the underlying data should be made freely available for researchers to use, wherever this is legal and ethical. Data availability allows validation, replication, reanalysis, new analysis, reinterpretation, or inclusion into meta-analyses, and facilitates reproducibility of research [1]. Making data available for all these uses provides a better “bang for the buck” out of scientific research, much of which is funded from public or nonprofit sources. Ultimately, our viewpoint is quite simple: Ensuring access to the underlying data should be an intrinsic part of the scientific publishing process.

## Developing a New Data Policy

Since their inception PLOS journals have requested that data be available, but we believe that providing more specific instructions for authors regarding appropriate data deposition options, and providing more information in the published article regarding how to access data, are important for both general readers and for research users of the research we publish. As a result, PLOS posted a revised Data Policy for comment on December 12, 2013 [2], and we are implementing the policy (Box 1) as of March 1, 2014. Authors of all research articles submitted to any PLOS journal on or after March 1 are required to include a statement detailing the availability of all data discussed in the manuscript.

**Box 1. PLOS Data Policy**

*In effect beginning March 1, 2014*

PLOS journals require authors to make all data underlying the findings described in their manuscript fully available without restriction, with rare exception^1^.

When submitting a manuscript online, authors must provide a *Data Availability Statement* describing compliance with PLOS's policy. The data availability statement will be published with the article if accepted.

Refusal to share data and related metadata and methods in accordance with this policy will be grounds for rejection. PLOS journal editors encourage researchers to contact them if they encounter difficulties in obtaining data from articles published in PLOS journals. If restrictions on access to data come to light after publication, we reserve the right to post a correction, to contact the authors' institutions and funders, or in extreme cases to retract the publication.

Methods acceptable to PLOS journals with respect to data sharing are listed below, accompanied by guidance for authors as to what must be indicated in their data availability statement and how to follow best practices in reporting [3]. If authors did not collect data themselves but used another source, this source must be credited as appropriate.

Authors who have questions or difficulties with the policy, or readers who have difficulty accessing data, are encouraged to contact the relevant journal office or data@plos.org.

**Acceptable data-sharing methods**

***Data deposition (strongly recommended):*** All data and related metadata underlying the findings reported in a submitted manuscript should be deposited in an appropriate public repository^2^, unless already provided as part of the submitted article. Repositories may be either subject-specific (where these exist) and accept specific types of structured data, or generalist repositories that accept multiple datatypes, such as Dryad [4]. Guidance on acceptable repositories is included below^2^. The *Data Availability Statement* must specify that data are deposited publicly and list the name(s) of repositories along with digital object identifiers or accession numbers for the relevant datasets. In some cases authors may not be able to obtain DOIs or accession numbers until the manuscript is accepted; in these cases, the authors must provide these numbers at acceptance. In all other cases, these numbers must be provided at submission.

***Data in supporting information files:***

For smaller datasets and certain data types, authors may upload data as supporting information files [5] accompanying the manuscript. Authors should take care to maximize the accessibility and reusability of the data by selecting a file format from which data can be efficiently extracted (for example, spreadsheets are preferable to PDF when providing tabulated data).

 

If data deposition or provision in supporting information is not ethical or legal (e.g., underlying data pose privacy or legal concerns, or include human participants^3^), the following two methods may be acceptable alternatives, subject to case-by-case evaluation:

***Data made available to all interested researchers upon request:*** *Data Availability Statement* must specify “Data available on request” and identify the group to which requests should be submitted (e.g., a named data access committee or named ethics committee). The reasons for restrictions on public data deposition must also be specified. Note that it is not acceptable for the authors to be the sole named individuals responsible for ensuring data access.

***Data available from third party:*** In the case of a primary dataset that was not originally generated by the authors of the submitted manuscript, appropriate data sharing may require that interested researchers obtain third-party data independently from the named original source. In this case, the *Data Availability Statement* must state the source of the data with full citation and, if the dataset cannot be provided, indicate “Data available from (named source).” The reasons for restrictions on public data deposition must also be specified.

**Unacceptable data access restrictions**

PLOS journals will not consider manuscripts where the following factors influence ability to share data:

- Authors will not share data because of personal interests, such as patents or potential future publications.
- The conclusions depend solely on the analysis of proprietary data (e.g., data owned by commercial interests). If proprietary data are used, the manuscript must include an analysis of public data that validates the conclusions so others can reproduce the analysis and build on the findings.

*^1^ Definition of data that must be shared*

PLOS defines the “minimal dataset” to consist of the dataset used to reach the conclusions drawn in the manuscript with related metadata and methods, and any additional data required to replicate the reported study findings in their entirety. Core descriptive data, methods, and study results should be included within the main paper, regardless of data deposition. PLOS does not accept references to “data not shown”. Authors who have datasets too large for sharing via repositories or uploaded files should contact the relevant journal for advice.

*^2^ Guidance on data repositories*

PLOS requires that authors comply with field-specific standards for preparation and recording of data [6] and to select repositories appropriate to their field, for example deposition of microarray data in ArrayExpress or GEO; deposition of gene sequences in GenBank, EMBL or DDBJ; and deposition of ecological data in Dryad [7]. Authors are encouraged to select repositories that meet accepted criteria as trustworthy digital repositories, such as criteria of the Centre for Research Libraries [8] or Data Seal of Approval [9]. Large, international databases are more likely to persist than small, local ones. Copyright licensing for data held in repositories may be unclear. If authors use repositories with stated licensing policies; the policies should not be more restrictive than CC-BY.

*^3^ Guidance on sharing datasets that derive from clinical studies or other work involving human participants*

For studies involving human participants, data must be handled so as to not compromise study participants’ privacy. PLOS recommends that researchers follow established guidance and applicable local laws in ensuring they do not compromise participant privacy. Resources which researchers may consult for guidance include: US National Institutes of Health: Protecting the Rights and Privacy of Human Subjects [10], Canadian Institutes of Health Research Best Practices for Protecting Privacy in Health Research [11], UK Data Archive: Anonymisation Overview [12], Australian National Data Service: Ethics, Consent and Data Sharing [13]. Steps necessary to protect privacy may include de-identification, blocking portions of the database, or license agreements directed specifically at privacy concerns. Authors should indicate, as part of the ethics statement, the ways in which the study participants’ privacy was preserved. If license agreements apply, authors should note the process necessary for other researchers to obtain a license.

This policy was developed after extensive consultation with PLOS Editors in Chief, in-house professional editors, and Academic Editors, who include practicing scientists from a variety of disciplines. We also appreciate input on the policy from others during the comment period, via Twitter and email. The feedback helpfully identified points for clarification, but the policy remains unaltered.

## Policy Clarification

One point for clarification is the issue of “data available on request.” We strongly believe that data should be freely available all the time without having to go through a gatekeeper, but we recognize that in some instances patient privacy or other concerns may preclude making data freely available to all. If ethical barriers prevent complete data release, authors should adhere to the guidelines noted in the Data Policy, which requires authors to identify a named ethics or data access committee, or other equivalent group, that can provide access. We appreciate that such committees may not already exist, and if that is the case, please note details of your situation when submitting to PLOS. We are still investigating potential solutions to this issue and until we have determined a standard course of action, we will work with authors for whom this presents a challenge.

A second point to clarify is that the Data Policy states the “minimal dataset” consists “of the dataset used to reach the conclusions drawn in the manuscript with related metadata and methods, and any additional data required to replicate the reported study findings in their entirety.” We want to be clear that this does not mean that authors must submit their entire dataset, or absolutely all raw data collected during an investigation, but that they must provide the portion that is relevant to the specific study.

## Implementation

As of March 1, 2014, authors submitting their research manuscript to PLOS journals will find a field in the online submission form where they will be asked to provide the data availability information, which will then be available to editors and reviewers during the review process and, in the event of publication, will be published with the article. Research studies that are submitted March 1, 2014 or later, and are published, will thus all include such a data availability statement. Authors may choose to include a published data availability statement for articles submitted before this cut-off, subject to practical considerations.

## Conclusion

The intent of the PLOS Data Policy is to facilitate data availability and transparency. We encourage authors with questions or concerns to contact the individual PLOS journal, or the Data Policy team at data@plos.org. An FAQ is being developed and we will continue to add to it, and in response to feedback and experience we may choose to further revise the Data Policy itself. We encourage your submissions and look forward to better and more open data availability to help foster scientific progress and support research transparency.
